# Clinical Significance of Bone Metastases in Pleural Mesothelioma

**DOI:** 10.1111/1759-7714.70033

**Published:** 2025-03-11

**Authors:** Mitsutomi Miyake, Kozo Kuribayashi, Hiroshi Doi, Aki Kubota, Taiichiro Otuski, Yoshiki Negi, Koji Mikami, Ryo Takahashi, Akifumi Nakamura, Yasuhiro Nakajima, Daichi Fujimoto, Kazuhiro Kitajima, Toshiyuki Minami, Takashi Kijima

**Affiliations:** ^1^ Department of Respiratory Medicine and Hematology Hyogo Medical University Nishinomiya Japan; ^2^ Department of Radiation Oncology, Faculty of Medicine Kindai University Osakasayama Japan; ^3^ Division of Thoracic Surgery, Department of Surgery Hyogo Medical University Nishinomiya Japan; ^4^ Department of Radiology Hyogo Medical University Nishinomiya Japan

**Keywords:** bone metastasis, keynote 483, pleural mesothelioma, prognosis, skeletal‐related events

## Abstract

**Background:**

Bone metastasis (BoM) is common in advanced cancer, but its incidence in pleural mesothelioma (PM) remains unclear. This study aimed to determine the incidence of BoM in PM patients and assess its prognosis and risk factors to clarify its clinical significance.

**Methods:**

A retrospective analysis was conducted on 515 histologically confirmed PM patients enrolled between January 2011 and December 2020. The cumulative incidence of BoM was calculated using the Kaplan–Meier method, with group differences assessed via log‐rank tests. Risk factors for BoM were evaluated using multivariate logistic regression.

**Results:**

The median follow‐up was 13.3 months (range: 0.2–106.7 months). BoM was detected in 59 patients (11.5%) at diagnosis or during disease progression. Multivariate analysis identified non‐epithelial histology (odds ratio [OR]: 2.189, 95% confidence interval [CI]: 1.179–4.065, *p* = 0.013) as an independent risk factor for developing BoM. Patients with BoM had worse overall survival (OS) compared to those without BoM (median OS: 18.6 months vs. 21.7 months, *p* = 0.03).

**Conclusions:**

BoM in PM occurs less frequently than in primary lung cancer, with non‐epithelial histology being more commonly associated with BoM. Patients with BoM had a poor prognosis, particularly when BoM was present at diagnosis. This study is limited by its retrospective design, which may introduce biases related to data collection and patient selection. Future prospective studies are needed to validate these findings.

## Graphical abstract

Bone metastasis (BoM) was observed in 11.5% of pleural mesothelioma (PM) patients, with non‐epithelial histology being an independent risk factor for developing BoM. Patients with BoM had significantly worse survival outcomes compared to those without BoM.

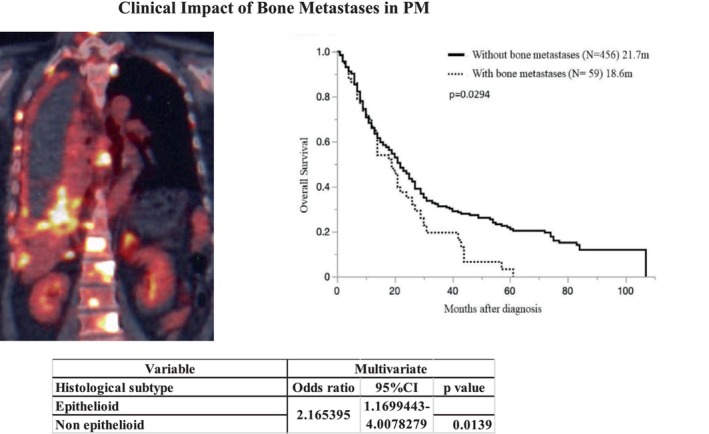

## 1 Introduction

Pleural mesothelioma (PM) is a rare and aggressive primary pleural neoplasm associated with asbestos exposure [1]. In August 2018, the Pharmaceuticals and Medical Devices Agency (PMDA) in Japan granted world‐first approval for nivolumab, an immune checkpoint inhibitor (ICI), as monotherapy for previously treated unresectable advanced or recurrent pleural mesothelioma (PM) [2]. This approval marked the first time in the world that nivolumab could be used as a treatment option for previously treated PM, which had lacked effective therapies until then. Subsequently, as if following Japan's lead, in October 2020, the FDA approved a combination therapy of nivolumab and ipilimumab (CheckMate743 Regimen: ICI Dual Therapy) as the world's first and only immunotherapy for untreated unresectable malignant pleural mesothelioma (MPM), establishing it as a new standard first‐line treatment for PM for the first time in approximately 15 years [3, 4]. However, despite these advancements, most patients are already unresectable at the time of diagnosis, resulting in a poor prognosis with a median survival of approximately 12 months [5].

PM is known to remain confined to the thoracic cavity for a relatively long period, with extensive invasion into adjacent structures such as the mediastinum and diaphragm. However, distant metastases are relatively rare, and reports quantifying the frequency of distant metastases in PM are scarce [6]. Among malignant tumors, bone metastases (BoM) are frequently observed in cancers such as lung cancer (30%–40%), prostate cancer (65%–75%), and breast cancer (65%–75%), whereas the incidence is low in hepatocellular carcinoma (2%–12.9%) [7]. When BoM progresses, it results in severe bone pain, pathological fractures, spinal cord compression, and hypercalcemia, significantly impacting both prognosis and quality of life (QOL). These symptoms, known as skeletal‐related events (SREs), are considered poor prognostic factors for malignant tumors [8].

In recent years, advancements in imaging techniques for detecting BoM and improvements in systemic treatments have prolonged survival, potentially increasing the likelihood of cancer metastasizing to bones and elevating the incidence of BoM. However, the clinical characteristics and prognosis of BoM remain unclear.

Currently, with immunotherapy becoming the standard treatment for PM, the approval of the Keynote483 Regimen (Platinum/Pemetrexed/Pembrolizumab: Combination Immunotherapy) as a new first‐line therapy for PM is highly anticipated [9]. The introduction of this new regimen will undoubtedly raise critical questions about selecting the most appropriate immunotherapy regimen for PM, similar to the situation in lung cancer.

This single‐center retrospective study aims to evaluate the incidence, risk factors, and survival outcomes of BoM, including SREs, throughout the clinical course of PM. Additionally, the study investigates whether the presence of distant metastases reflects disease progression in PM and examines the potential role of these findings in guiding the choice between ICI Dual Therapy (CheckMate743) and Combination Immunotherapy (Keynote483) as first‐line treatments for PM.

## 2 Methods

### 2.1 Patient Population

In this retrospective study, 515 patients (389 epithelial types, 73 sarcoma types, 42 biphasic types, and 11 desmoplastic types) were diagnosed with PM histopathologically at the time of diagnosis confirmation and afterward between January 2011 and December 2020 at the Department of Respiratory Medicine and Hematology, Hyogo Medical University, were analyzed. Fifty‐nine patients with BoM were confirmed during the clinical course of the MPM disease. Baseline demographic and clinicopathological variables were collected retrospectively from patients' medical records.

These included age at initial diagnosis, gender, histological subtype, clinical stage, and baseline Eastern Cooperative Oncology Group (ECOG) performance status (PS). Clinical staging was determined according to the International Mesothelioma Interest Group (IMIG) staging system [10].

BoM diagnosis was confirmed using fluorodeoxyglucose‐positron emission tomography (FDG‐PET) and other imaging systems (X‐ray, computed tomography [CT], magnetic resonance imaging [MRI], etc.). However, a histological diagnosis from BoM sites was not always performed. Rib fractures due to direct invasion of the chest wall and spinal fractures due to direct invasion of the spine were excluded.

SREs were defined as pathological fractures, radiation therapy for bone lesions, surgical operations for bone lesions, spinal cord compression, and hypercalcemia associated with a malignant tumor. We analyzed PMs along with SREs.

This study was approved by the institutional review board of Hyogo Medical University (approval number: 202209‐042). Patient confidentiality was strictly maintained. The requirement for informed consent was waived for this retrospective study.

### 2.2 Patient Selection and Flow Diagram (Figure 1)

**FIGURE 1:**
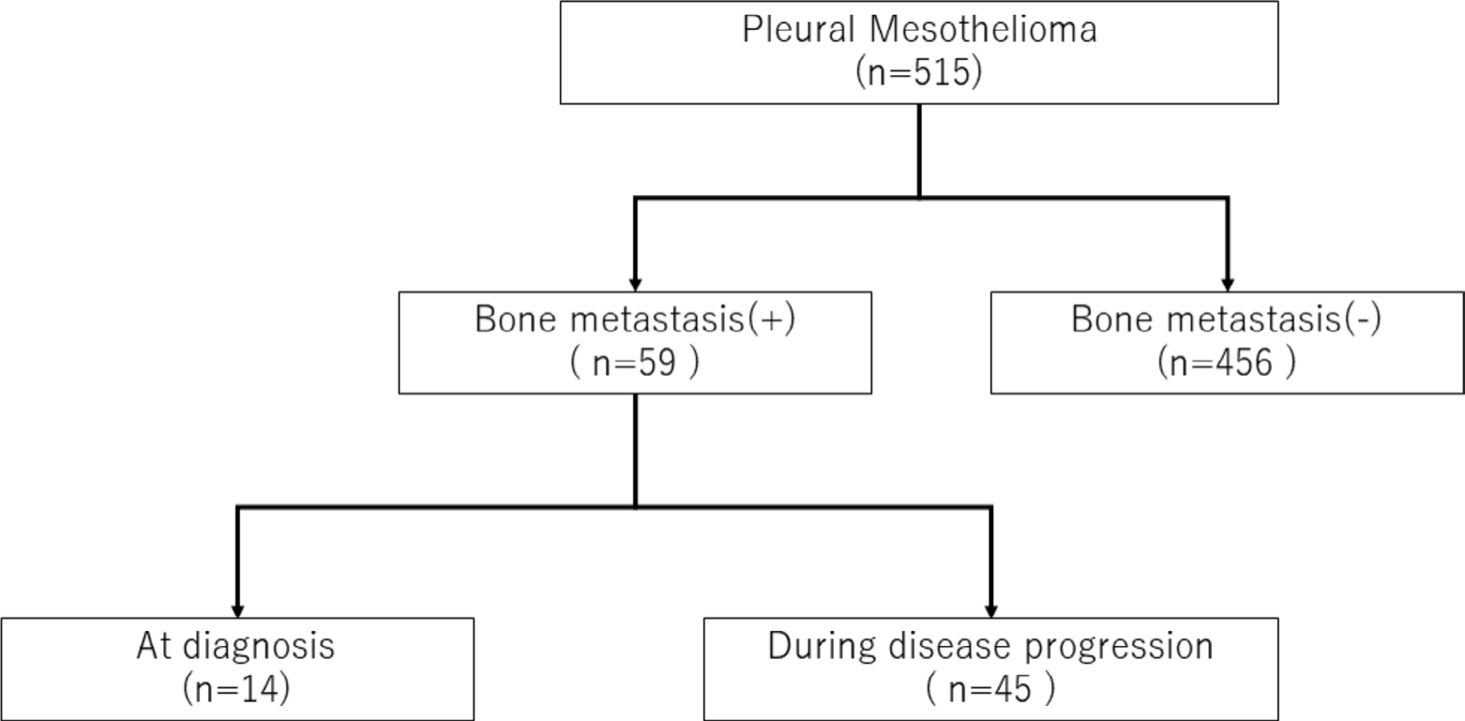
Patient selection and flow diagram.

To enhance transparency in patient selection and study procedures, we created a detailed patient flow diagram. This diagram outlines the process from the initial screening of eligible participants to the final inclusion in the analysis. Specifically, it illustrates the criteria used for patient selection, the exclusion of ineligible cases, and the categorization of patients into study groups based on their clinical characteristics and outcomes. This addition aims to provide a clear and comprehensive overview of the study design and to address potential concerns regarding selection bias.

### 2.3 Pain Assessment

In this study, pain assessment was conducted using patient‐reported outcomes, relying on qualitative descriptions provided by the patients during clinical evaluations. No standardized quantitative measures, such as visual analog scales (VAS) or numerical rating scales (NRS), were employed. This approach, while reflective of patient experiences, may limit the ability to perform precise comparisons or statistical analyses. To enhance transparency, we have acknowledged this limitation in the study's Limitations section.

### 2.4 Statistical Analysis

A comparison of patient characteristics at diagnosis was performed using Fisher's exact test or Student's *t*‐test, as appropriate. Overall survival (OS) was calculated from the date of initial diagnosis to the date of last follow‐up or death from any cause using Kaplan–Meier methods. Inter‐group differences were compared with the log‐rank test. To determine risk factors associated with the development of BoM, univariate and multivariate logistic regression models with a stepwise selection procedure were generated. The two‐tailed *p*‐value < 0.05 was considered statistically significant. All statistical analyses were performed with JMP Pro (version 14.2.01; SAS Institute, USA).

## 3 Results

### 3.1 Patient Characteristics

Five hundred fifteen patients were followed up for 13.3 months (median follow‐up, 0.2–106.7 months). Fifty‐nine patients developed BoM, 14 at the time of MPM diagnosis, and 45 during the clinical course of PM. The crude incidence of BoM was 2.7% at diagnosis and 11.5% overall. At the time of BoM detection, 36 patients (62.7%) had adrenal (*n* = 7), lung (*n* = 15), lymph node (*n* = 17), liver (*n* = 8), muscle/soft tissue (*n* = 2), and other distant metastasis, including brain metastasis (*n* = 5). The 1‐ and 2‐year cumulative BoM rates were 40.0% and 84.4%, respectively. The baseline characteristics of the patients with BoM (*n* = 59) are presented in Table 1.

**TABLE 1:** Characteristics of patients with bone metastasis.

	*N*	%
Median age (range)	68 (44–80)	
Gender		
Male	46	78.0
Female	13	22.0
Histology		0.0
Epithelioid	38	64.4
Sarcomatiod	17	28.8
Biphasic	4	6.8
Treatment for primary tumor		0.0
Chemotherapy	41	69.5
Multimodality therapy	14	23.7
BSC	4	6.8
Interval to BoM		
Median (range)	15.3 M (0–44.3 M)	

In the BoM (+) patient cohort (*n* = 59), the median age (range) was 68 years (44–80), with 46 males (78%), and 13 females (22%). There were 38 epithelial types (64.4%), 15 sarcoma types (25.4%), four biphasic types (6.8%), and two fibrogenic types (3.4%) cases. In sites such as the spine, pelvis, ribs, etc., which have a lot of red marrow, many BoMs were observed. The median time to onset of BoM from the initial diagnosis was 15.3 months (0–44.3 months).

The clinicopathological characteristics of the patients with BoM were compared with those of patients without BoM (non‐BoM) (Table 2). Regarding PS and treatment modality, no difference was observed between the two groups. Patients with BoM were treated with systemic chemotherapy (*n* = 41), combined modalities (*n* = 14), and best supportive care (*n* = 4). There was no difference between the two groups with respect to age at diagnosis. BoM was frequently observed in the nonepithelial type of histological subtype (*p* = 0.0346) and Stage I–II of IMIG Stage (*p* = 0.0349).

**TABLE 2:** Patient characteristics.

Characteristic	Bone metastasis (*n* = 59)	Non‐bone metastasis (*N* = 456)	*p*
Median age, years	68	69	0.4572
Men	46 (78%)	381 (84%)	0.2834
ECOG PS > 2	7 (12%)	25 (5%)	0.056
Histological subtype			0.0346
Epithelioid	38 (64%)	351 (77%)	
Nonepithelioid	21 (36%)	105 (23%)	
IMIG stage			0.0349
1 ~ 2	30 (51%)	296 (65%)	
3 ~ 4	29 (49%)	160 (35%)	
Treatment modality			0.8412
Multimodality therapy	14 (24%)	94 (21%)	
Chemotherapy	41 (69%)	333 (73%)	
Best supportive care	4 (7%)	29 (6%)	

### 3.2 Survival Curve

**FIGURE 2:**
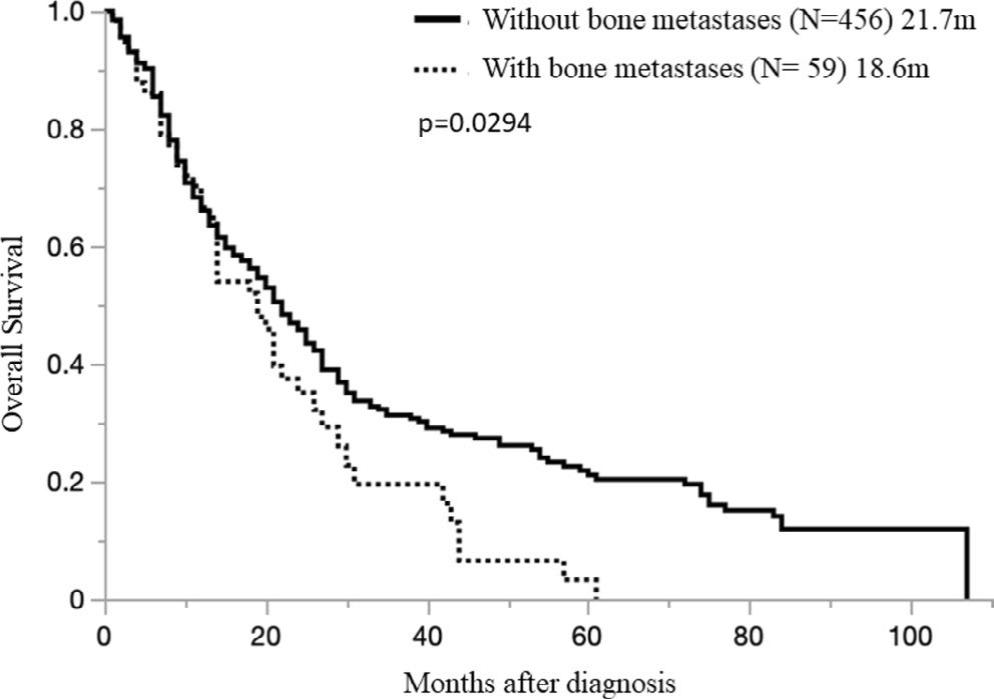
Overall survival in pleural mesothelioma patients with and without bone metastases.

Among the non‐BoM group (456 cases; OS, 21.7 months [0.2–106.7 months]) and the BoM group (59 cases; OS 18.6 months 0.5–60.8 months), the BoM group had a poor prognosis (*p* = 0.0294) (Figure 2). In the BoM group, the median survival time after onset was 4.03 months (0.4–34.9 months).

**FIGURE 3:**
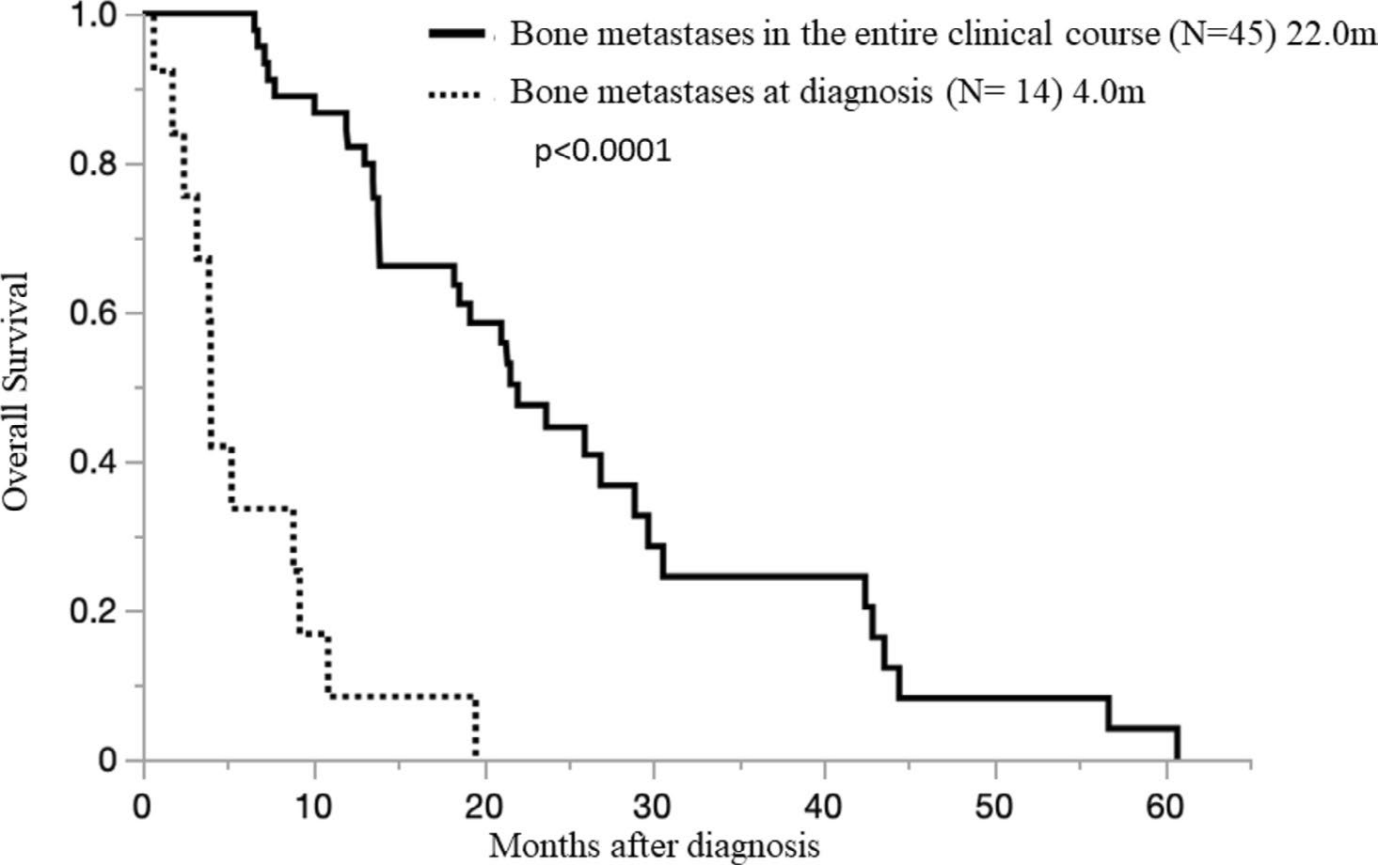
Overall survival in pleural mesothelioma patients with bone metastases in the entire clinical course and at diagnosis.

**FIGURE 4:**
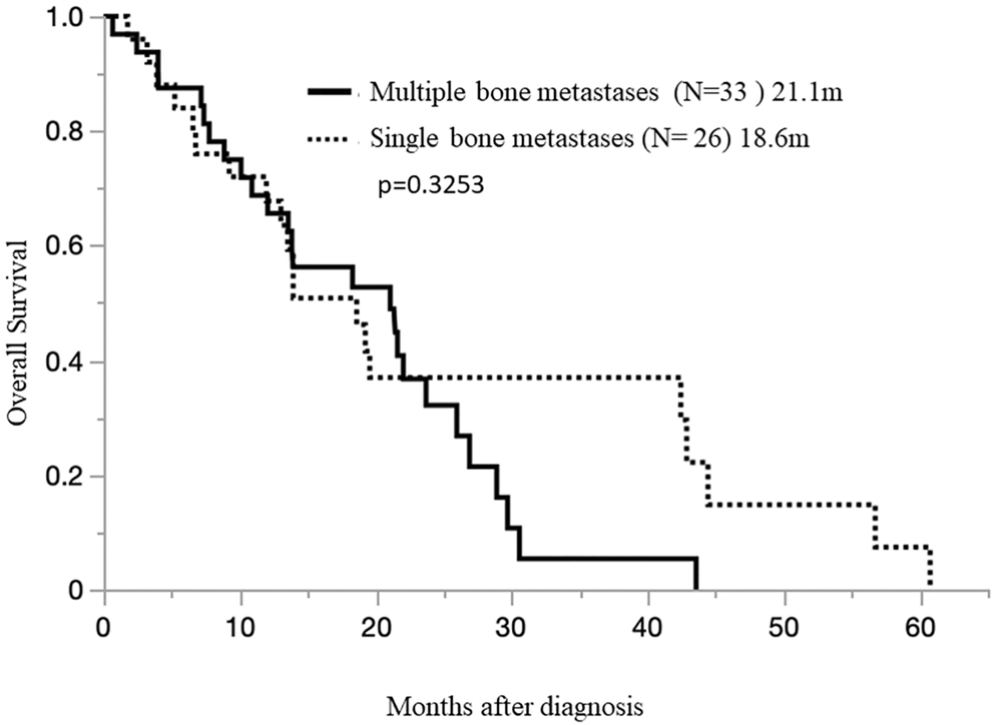
Overall survival in pleural mesothelioma patients with multiple bone metastases and those with a single bone metastasis.

Then the time of appearance of BoM was examined. Among the BoM at diagnosis (+)group (14 cases; OS median 4.0 months [0.5–19.6 months]) and the BoM during the course (+)group (45 cases OS 22.0 months [6.5–60.8 months]), the BoM at diagnosis group had a worse prognosis (*p* < 0.0001) (Figure 3). Furthermore, we examined the number of BoMs. Among the single BoM site group (26 cases; OS 18.6 months [0.7–60.8 months]) and the multiple site BoM group (33 cases; OS 21.1 months [0.5–21.6 months]), no difference in survival was observed between the groups due to the number of BoM (*p* = 0.3253) (Figure 4).

**FIGURE 5:**
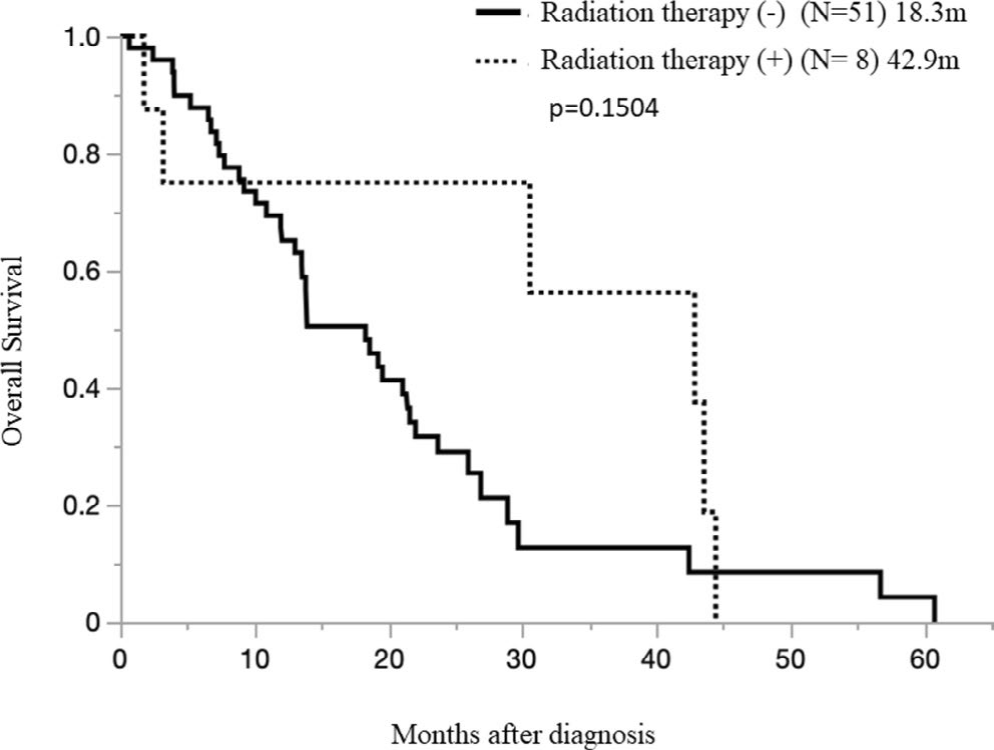
Overall survival in pleural mesothelioma patients with bone metastases, with or without radiation therapy.

We examined the effect of radiotherapy on BoM. Among the RT(+)group (8 cases; OS 42.9 months [1.8–44.4 months]) and RT(−)group (51 cases; 18.3 months [0.5–60.8 months]) BoM, the difference was nonsignificant (*p* = 0.3253) (Figure 5). Although there was a tendency for the survival period to be prolonged in the group that received radiotherapy.

**FIGURE 6:**
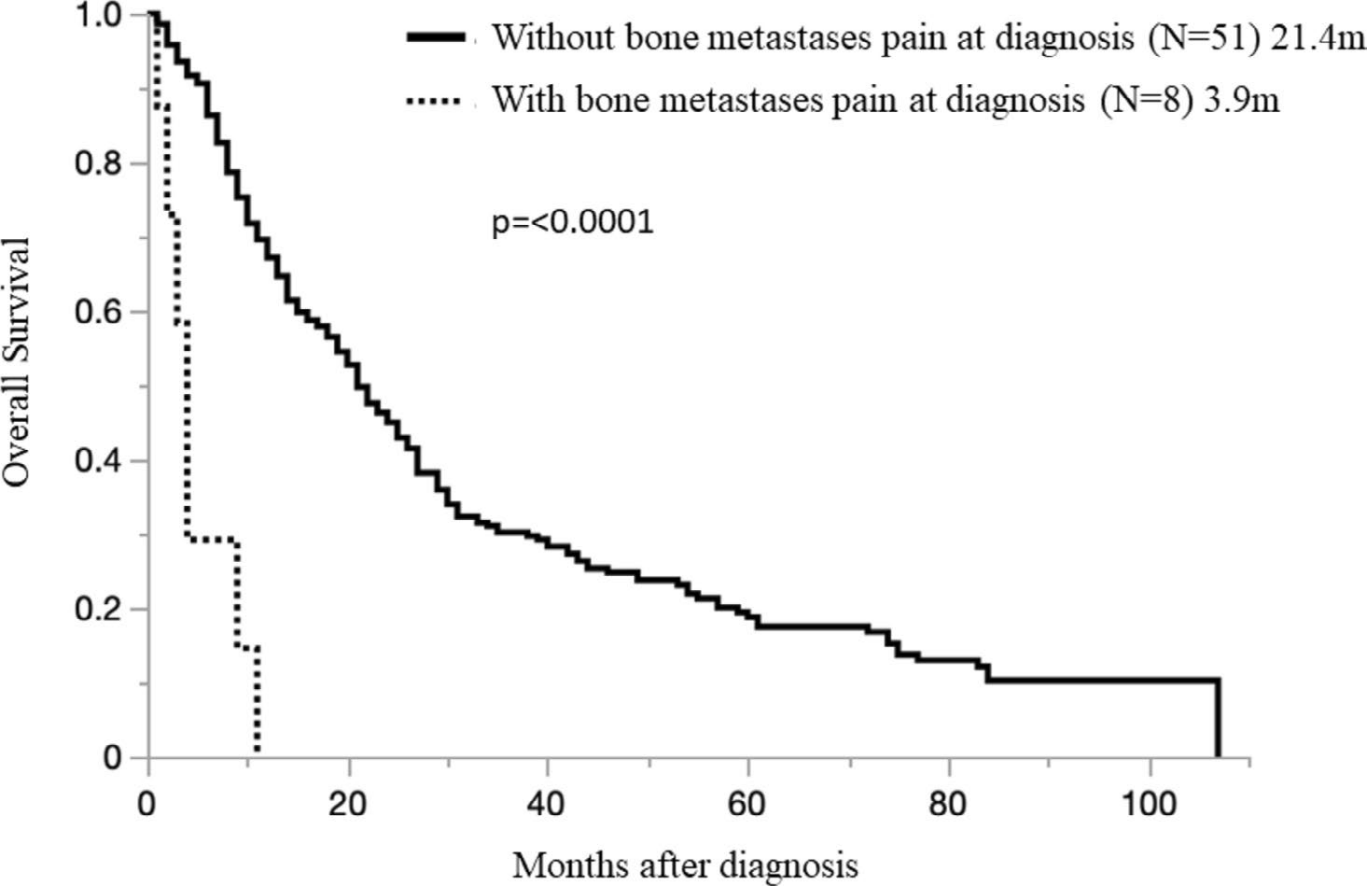
Overall survival in pleural mesothelioma patients with bone metastases, with or without bone pain.

In examining the presence or absence of pain due to BoM, the bone pain (+) case group (8 cases; OS 3.9 months [0.5–10.9 months]) and the bone pain (−) case group (51 cases; OS 21.4 months [0.5–56.7 months]), the prognosis was worse in the group with pain (*p* < 0.0001) (Figure 6).

### 3.3 SREs Analysis

**FIGURE 7:**
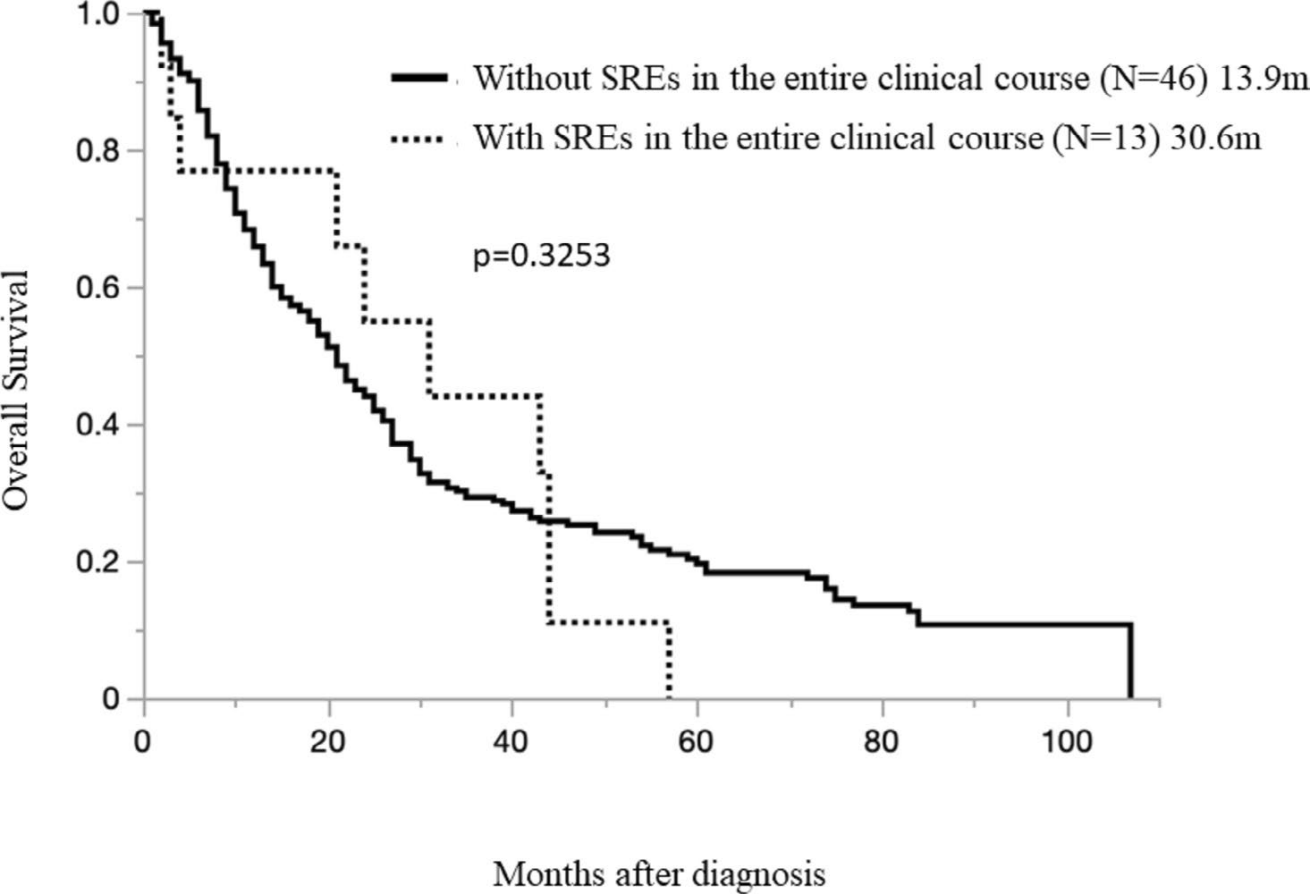
Overall survival in pleural mesothelioma patients with bone metastases, with or without SREs during the entire clinical course.

There were 59 cases of PM with BoM in all clinical courses. SRE occurred in 13 patients (2.52%) of the 59 PMs, and the details are shown in Table 3. Among the SREs (+) case group (13 cases; OS 30.6 months [1.8–56.7 months]) and SREs (−) case group (46 cases; OS, 13.9 months [0.7–60.8 months]) there was no difference in survival between the presence or absence of SREs (*p* = 0.0839) (Figure 7).

**TABLE 3:** Cox regression model for factors associated with the incidence of bone metastases.

Variable	Univariate			Multivariate		
	Odds ratio	95% CI	*p*	Odds ratio	95% CI	*p*
Age at initial diagnosis						
> 65 years	1.638	0.9236–2.9072	0.08			
≧ 65 years	1					
ECOG PS						
1–2	0.430892	0.177634–1.045223	0.056			
> 2	1					
Histological subtype						
Epithelioid	1					
Non epithelioid	1.847368	1.03869–3.285647	0.0259	2.165395	1.1699443–4.0078279	0.0139
IMIG stage						
1–2	1					
3–4	1.788333	1.036499–3.085518	0.0349	1.7385293	0.9646578–3.1332191	0.0657
Treatment modality						
Surgery+	1					
Surgery−	0.834649	0.439543–1.584915	0.343			

PMs along with SREs analysis showed pathological fracture (7 cases, 1.36%); radiation therapy for bone lesions (8 cases, 1.55%); surgical operation for bone lesions (3 cases, 0.58%); spinal cord compression (6 cases, 1.17%); and hypercalcemia associated with malignant tumors (1 case, 0.19%).

### 3.4 Risk Factors

Univariate analysis showed non‐epithelial histology (OR 1.847; 95% Confidence interval [CI] 1.039–3.286, *p* = 0.0284) and IMIG stage III‐IV (OR 1.788, 95% CI 1.036–3.086, *p* = 0.0259); however, BoM was found to be a potential risk factor. Furthermore, the multivariate analysis showed non‐epithelial tissue type (OR 2.189, 95% CI 1.179–4.065, *p* = 0.013) and that BoM was associated with a higher risk (Table 3).

## 4 Discussion

PM is a malignant chest tumor, with a prevalence of approximately 1/70, which is rarer than lung cancer. It stays in the thoracic cavity relatively long and affects the mediastinum, diaphragm, pleural cavity, and surrounding organs. PM is known to have malignant local infiltration, but relatively few distant metastasis [11]. To the best of our knowledge, this is the first report to focus on BoM incidence, risk factors, and survival outcomes in patients with PM.

In our study cohort, 11.5% (59/515) of patients with PM had BoM. In a previous report, the postmortem examination of 318 PM cases revealed distant metastasis in 54% of cases, with BoM in only 0.63% (2/318) [12]. Whereas in a retrospective radiological evaluation of 164 patients with PM, 67% (110/164) had distant metastasis, including bone (19%), visceral (14%), contralateral lung (35%), and peritoneal metastasis (22%), the most common sites of distant metastasis [13]. Based on the above, the frequency of BoM in PM has varied in past reports (0.63%–19%); however, in any case of lung cancer, the frequency of BoM (30%–40%) was lower. Our study showed that the outcome of BoM was equally poor regardless of the treatment method; therefore, BoM should not be overlooked in patients with PM.

Furthermore, our study clarified that the prognosis of PM cases with BoM was worse than that of PM cases without BoM. That is, even among MPM cases with BoM diagnosis at the beginning (+) case group (14 cases) and BoM during the course (+) case group (45 cases), OS was 4.0 months (0.5–19.6 months) and 22.0 months (6.5–60.8 months), respectively. The prognosis was significantly worse in the group with BoM at diagnosis (*p* < 0.0001) (Figure 3).

These findings are of critical importance and suggest that cases with bone metastasis at diagnosis clearly have the worst prognosis in each group; therefore, caution should be exercised. Additionally, the group in which BoM appeared during the course (OS 22.0 months [6.5–60.8 months]) OS was relatively longer than the BoM at diagnosis group (456 cases; OS, 21.7 months [0.2–106.7 months]), which may be because BoM appeared during the course.

In this study, while there were clearly symptomatic cases of bone metastases (8 cases), it cannot be denied that among the remaining cases (51 cases), there may have been a significant number of patients without bone pain, likely asymptomatic. In clinical practice, despite the limitations in detection circumstances, identifying the characteristics of such patient subsets would be highly valuable, particularly from a quality‐of‐life perspective.

We have previously reported on the utility of FDG‐PET in assessing treatment response in pleural mesothelioma [14] and identifying biopsy sites in peritoneal mesothelioma [15]. Additionally, prior studies have demonstrated that FDG‐PET/CT is a highly valuable tool in the management of malignant pleural mesothelioma, particularly for facilitating comprehensive disease evaluation [16]. Moving forward, it will be essential to assess the role of FDG‐PET as a highly sensitive screening tool for the early detection of asymptomatic distant metastases, including bone metastases, in pleural mesothelioma. Insights from such investigations should be leveraged to optimize treatment selection for mesothelioma immunotherapy.

Our study also investigated the involvement of SREs in the course of PM, which has not been reported previously. In general, the PS of patients with SREs is extremely poor in patients with lung cancer, etc., and it has a serious impact on the treatment plan. Symptomatic treatment for pain relief is preferred for NSCLC with bone metastasis with severe symptoms [17]. In fact, the most common SREs in our study were radiotherapy for bone lesions. Radiotherapy plays an important role not only in the prevention of fractures due to bone metastasis and the avoidance of spinal paralysis but also in the treatment of pain relief. However, the effect of symptomatic treatment on survival time is limited. In fact, the median OS of patients with SREs (+) (13 cases) and patients with SREs (−) (46 cases) was 30.6 months (range, 1.8–56.7 months) and 13.9 months (range, 0.7–60.8 months), respectively, showing no difference in survival time between patients with and without SREs (*p* = 0.3253) (Figure 7).

Pain is one of the clinical signs in PM, which has a special meaning in it. Even in respectable cases, pain is of utmost importance, as surgery is not recommended in patients with uncontrolled pain [18]. Therefore, in this study, we investigated the effects of the presence or absence of pain caused by BoM in the bone pain (+) group (28 cases; OS 21.4 months [0.5–56.7 months]) and no bone pain (−)group (31 cases; 16.2 months [0.7–60.8 months]). Our results showed no difference in survival due to BoM pain (*p* = 0.3253) among the groups. However, the time of onset of bone pain in the bone pain (+) group (8 cases; OS, 3.9 months [0.5–10.9 months]) and progressing bone pain (+) group (20 cases; OS, 23.7 months [4.0–56.7 months]) showed that the prognosis was worse in the group with bone pain (*p* < 0.0001) (Figure 6). The findings of this study suggest that pain control for bone metastases may influence survival outcomes. However, it is also possible that the presence of bone pain itself serves as a marker of advanced disease progression. Consequently, the impact of symptomatic bone metastases on survival may be confounded by this association. Determining whether pain control for bone metastases directly contributes to improved survival outcomes will require further investigation. This study highlights the necessity for such future research.

In fact, while immune checkpoint inhibitors (ICIs) have become the cornerstone of standard treatment for thoracic malignancies, including lung cancer and mesothelioma, there are no definitive guidelines on how to select between ICI dual therapy and combination immunotherapy. In mesothelioma, clinicians are often faced with the challenge of deciding whether to use CheckMate743 (ICI dual therapy) or Keynote483 (combination immunotherapy).

Our study highlights a novel finding: patients presenting with BoM at the time of initial diagnosis have a poorer prognosis compared to those who develop BoM during the disease course. This observation may provide a potential framework for addressing this treatment selection dilemma. Specifically, if distant metastases, such as BoM, reflect a greater disease burden, combination immunotherapy, which incorporates chemotherapy and offers potentially stronger disease control, could be more appropriate as a first‐line treatment for such patients.

Although further research is required to validate this hypothesis, our findings suggest that disease burden, including the presence of BoM, could influence the choice of immunotherapy regimen. Understanding how disease progression impacts treatment efficacy could provide a much‐needed foundation for establishing evidence‐based guidelines, ensuring that the most suitable therapeutic strategy is selected for each patient.

The histological subtype is one of the most important clinicopathological features of MPM that affects survival. Among the non‐epithelial types, sarcomatoid histology has the poorest prognosis, with outcomes reported as 5–6 months for sarcomatoid cases and 11.8 months for biphasic mesothelioma cases with more than 50% epithelioid component [19]. Our multivariate analysis showed that BoM was associated with a higher risk of non‐epithelial histology subtype (OR 2.189, 95% CI 1.179–4.065, *p* = 0.013) (Table 3). A second‐best approach to improve prognosis would be to identify patients with PM at high risk of developing BoM and perform periodic imaging studies even if the patient is asymptomatic.

The potential association between bone metastases (BoM) and other distant metastatic sites, such as the lungs, liver, or brain, remains an important area for further investigation. While our study did not specifically analyze these relationships, understanding metastatic patterns in pleural mesothelioma could inform targeted screening protocols and improve the early detection of BoM. Previous reports have investigated brain metastases in mesothelioma [6], highlighting the importance of exploring the relationships among various distant metastatic sites. For instance, patients presenting with specific metastatic patterns may benefit from more sensitive imaging modalities, such as FDG‐PET, to identify asymptomatic BoM.

Previous studies have highlighted the utility of FDG‐PET in evaluating distant metastases and guiding management decisions in mesothelioma [16]. Expanding its application to include early detection of BoM in specific patient subsets could enhance clinical decision‐making and help preserve quality of life (QOL) in patients with advanced disease. Future studies are needed to explore the interplay between BoM and other metastatic sites and to establish evidence‐based screening strategies tailored to mesothelioma's unique metastatic behavior.

Our study has several limitations. First, this is a single‐center, retrospective study with relatively small numbers of patients and events, which limited the statistical analysis. Second, most BoMs were diagnosed based on the radiological evaluation. Therefore, the possibility of extracting a false‐positive BoM cannot be completely denied.

Besides, a notable limitation of this study is the medium follow‐up period, which may not fully capture the long‐term progression of pleural mesothelioma or the eventual development of BoM. Longer follow‐up periods are essential to better understand the true incidence and timing of BoM, particularly in the context of evolving treatment strategies such as immunotherapy. Extended follow‐up would provide a more comprehensive evaluation of disease dynamics and skeletal‐related events, allowing for a more accurate assessment of prognostic factors and therapeutic outcomes. Future prospective studies with longer follow‐up durations are needed to address this limitation and further validate the findings presented here.

In conclusion, the prognosis of patients with PM, along with BoM, is poor. The occurrence of BoM severely impacts patients' quality of life and survival. Therefore, clinicians should perform careful screening for BoM, especially in patients with high risk. At the same time, depending on the difference in the time of the first appearance of the BoM in patients with PM, the disease prognosis may also differ.

## 5 Limitations of the Study

This study has several limitations that warrant acknowledgment. First, the single‐center design may have introduced selection bias, as the patient population and treatment approaches may not fully represent those of other institutions or regions. Additionally, the lack of a validation cohort further limits the reproducibility of our findings. The results presented in this study reflect the clinical characteristics and treatment practices specific to our institution, which may differ significantly from those in other medical settings or geographic regions. For example, disparities in access to advanced imaging modalities, treatment protocols, or patient demographics could have influenced the observed outcomes. To enhance reproducibility and confirm these findings, future studies should include diverse patient populations from multiple institutions and regions. Such multicenter studies would provide a broader perspective and allow for more generalizable and globally applicable conclusions.

Second, survivorship bias may have influenced the results, as patients who survived longer had a greater likelihood of developing bone metastases due to extended disease progression and follow‐up. This factor, combined with potential underreporting of asymptomatic cases, introduces detection bias. The identification of bone metastases in this study relied heavily on imaging studies, which may not capture all cases, particularly those without overt symptoms. These factors underscore the importance of prospective studies utilizing standardized and sensitive diagnostic protocols to mitigate such biases.

Third, the retrospective nature of the study carries inherent limitations. Retrospective analyses depend on the availability and completeness of medical records, which can lack uniformity and lead to inconsistencies in data collection. This design also precludes the ability to establish causation and limits the standardization of case selection and treatment approaches. To address these challenges, future research should employ prospective, multicenter designs with rigorous methodologies to validate and expand upon the findings presented here.

Finally, the underreporting of skeletal‐related events (SREs) due to the retrospective design is a notable limitation. Variability in clinical documentation and the absence of uniform reporting standards likely contributed to incomplete data regarding these events. Comprehensive prospective data collection is necessary to fully evaluate the impact of SREs on patient outcomes and treatment efficacy.

## Author Contributions

All authors had full access to the data in the study and take responsibility for the integrity of the data and the accuracy of the data analysis. Conceptualization: M.M. and K.K.; Data Curation: H.D., A.K., T.O.; Formal Analysis: H.D., A.K., T.O.; Funding acquisition: T.K.; Investigation: M.M., K.K., T.O., Y.N., K.M., R.T., A.N., Y.N., Ka.K.; Methodology: H.D., T.O.; Project administration: M.M.; Resources: Y.N., A.N.; Supervision: T.K.; Validation: D.F. and T.M.; Visualization: A.K., D.F., Ka.K., T.M.; Writing – original draft: K.K.; Writing – review and editing: M.M., K.K., H.D., A.K., T.O., Y.N., K.M., R.T., A.N., Y.N., D.F., Ka.K., T.M., T.K.

## Disclosure

The authors have no relevant financial or non‐financial interests to disclose.

## Ethics Statement

The study was approved by the Institutional Review Board of the Hyogo Medical University (approval number: 202209‐042) and was performed in accordance with the ethical standards as laid down in the 1964 Declaration of Helsinki and its later amendments or comparable ethical standards.

## Consent

The requirement for informed consent was waived for this retrospective study.

## Conflicts of Interest

Hiroshi Doi received speaker fees from AstraZeneca, Eisai, Boston Scientific Japan, Varian Medical Systems, and Accuray Japan K.K. Daichi Fujimoto has received grants and personal fees from AstraZeneca K.K. and Boehringer Ingelheim Japan Inc., as well as personal fees from Ono Pharmaceutical Co. Ltd., Bristol Myers Squibb Co. Ltd., Taiho Pharmaceutical Co. Ltd., Chugai Pharmaceutical Co. Ltd., MSD K.K., Eli Lilly Japan K.K., Daiichi Sankyo, Novartis Pharma K.K., Kyowa Kirin Co. Ltd., and Janssen Pharmaceutical K.K., outside the scope of the submitted work. The other authors have no conflicts of interest to declare.

## Data Availability

The datasets used and/or analysed during the current study are available from the corresponding author on reasonable request.
